# Meta-Apo improves accuracy of 16S-amplicon-based prediction of microbiome function

**DOI:** 10.1186/s12864-020-07307-1

**Published:** 2021-01-06

**Authors:** Gongchao Jing, Yufeng Zhang, Wenzhi Cui, Lu Liu, Jian Xu, Xiaoquan Su

**Affiliations:** 1 Single-Cell Center, CAS Key Lab of Biofuels, Shandong Key Lab of Energy Genetics and Shandong Institute of Energy Research, Qingdao Institute of BioEnergy and Bioprocess Technology, Chinese Academy of Sciences, Qingdao, China; 2 College of Computer Science and Technology, Qingdao University, Qingdao, China; 3 College of Control Science and Engineering, China University of Petroleum, Qingdao, China

**Keywords:** Microbiome, Metagenome, Amplicon, Function, Calibration

## Abstract

**Background:**

Due to their much lower costs in experiment and computation than metagenomic whole-genome sequencing (WGS), 16S rRNA gene amplicons have been widely used for predicting the functional profiles of microbiome, via software tools such as PICRUSt 2. However, due to the potential PCR bias and gene profile variation among phylogenetically related genomes, functional profiles predicted from 16S amplicons may deviate from WGS-derived ones, resulting in misleading results.

**Results:**

Here we present Meta-Apo, which greatly reduces or even eliminates such deviation, thus deduces much more consistent diversity patterns between the two approaches. Tests of Meta-Apo on > 5000 16S-rRNA amplicon human microbiome samples from 4 body sites showed the deviation between the two strategies is significantly reduced by using only 15 WGS-amplicon training sample pairs. Moreover, Meta-Apo enables cross-platform functional comparison between WGS and amplicon samples, thus greatly improve 16S-based microbiome diagnosis, e.g. accuracy of gingivitis diagnosis via 16S-derived functional profiles was elevated from 65 to 95% by WGS-based classification. Therefore, with the low cost of 16S-amplicon sequencing, Meta-Apo can produce a reliable, high-resolution view of microbiome function equivalent to that offered by shotgun WGS.

**Conclusions:**

This suggests that large-scale, function-oriented microbiome sequencing projects can probably benefit from the lower cost of 16S-amplicon strategy, without sacrificing the precision in functional reconstruction that otherwise requires WGS. An optimized C++ implementation of Meta-Apo is available on GitHub (https://github.com/qibebt-bioinfo/meta-apo) under a GNU GPL license. It takes the functional profiles of a few paired WGS:16S-amplicon samples as training, and outputs the calibrated functional profiles for the much larger number of 16S-amplicon samples.

## Background

**Fig. 1:**
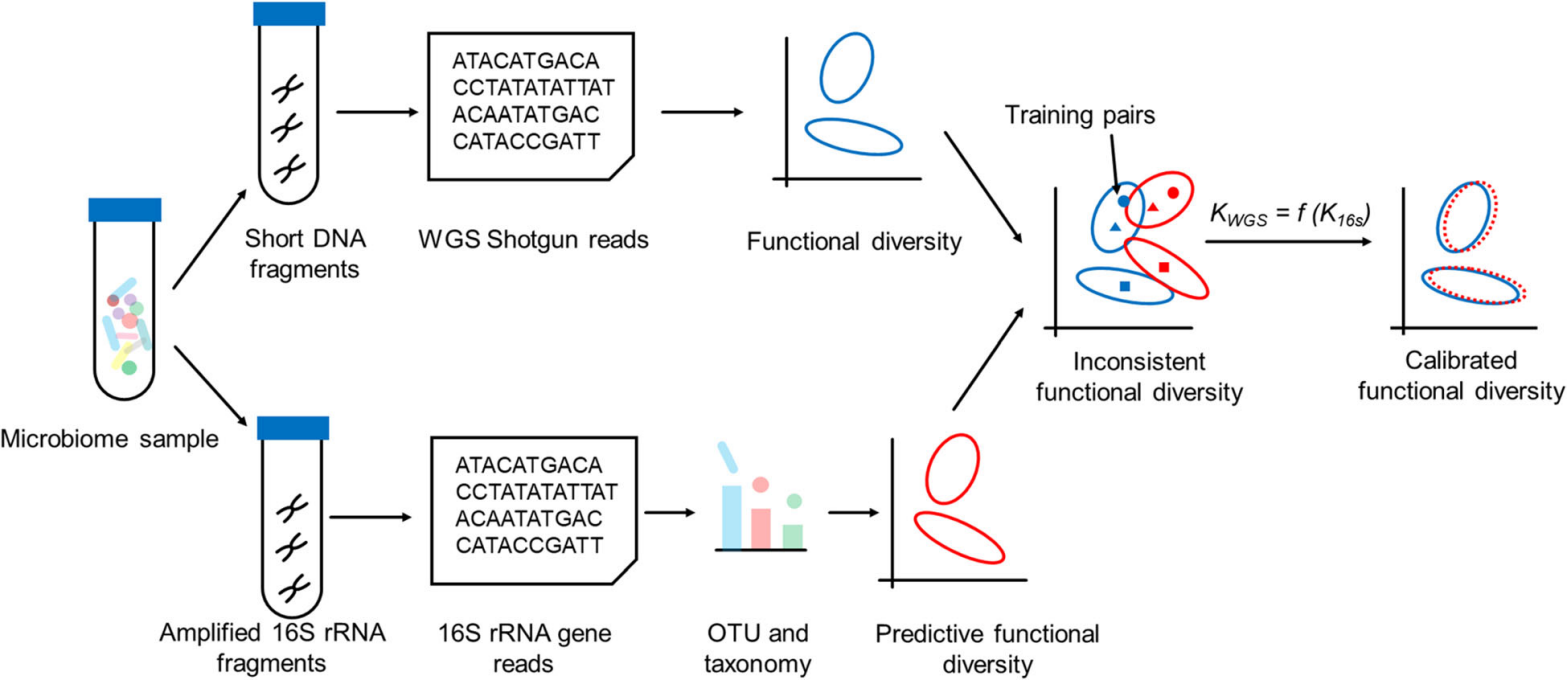
Calibration of predicted functional profiles of microbiome amplicon samples by a small number of WGS:16S-amplicon sample pairs for training

Interest in microbiome has been fueled by the ability to profile diverse microbial communities via high-throughput sequencing [1, 2], which generally adopt one of two strategies [3]: amplicon sequencing, which most often employs the 16S rRNA gene as a phylogenetic marker for bacteria, or shotgun whole-genome sequencing (WGS), which captures genome-wide sequences of the mixture of species within a sample. In amplicon sequencing, microbial taxonomy structure is revealed via PCR-based amplification using primers that target a specific region of the phylogenetic marker gene, however it does not directly yield the profile of functional genes. In contrast, shotgun WGS constructs a functional profile from metagenomic sequences [4], yet its broader application is limited by the much higher cost and complexity in both experiment and computation [3, 5, 6]. Therefore, computational tools that predict functional profile via 16S amplicons were introduced [7–10], e.g., PICRUSt derives diversity and relative abundance of molecular functions by tracing the sequenced 16S fragments to presently available microbial genomes. However, due to the amplification bias induced in 16S gene PCR [11, 12] and function profile variation among phylogenetically related genomes, microbiome functional profiles predicted from 16S amplicons can deviate greatly from WGS-derived ones (Fig. 1 and Fig. 3a).

To tackle this challenge, we present Metagenomic Apochromat (Meta-Apo). By training on only a small number of matched WGS:16S-amplicon data pairs (each pair is sequenced via both shotgun WGS and 16S-amplicon of the exactly same microbiome specimen), Meta-Apo produces for large-scale 16S-amplicon samples post-calibration functional profiles that are much more consistent with the WGS results (Fig. 1). Moreover, since shotgun WGS provides more stable microbiome-based disease detection across multiple studies than amplicons [13, 14], such calibration by Meta-Apo enables cross-platform functional comparison between WGS and amplicon samples and thus can greatly improve 16S-based microbiome diagnosis. For example, using 16S-derived functional profiles that are calibrated by WGS-derived functional profiles, gingivitis diagnosis accuracy was elevated to 95% from 65%. Therefore, Meta-Apo offers a low-cost strategy to obtain accurate and high-resolution view of microbiome functions based on primarily 16S amplicon data.

## Results

### Functional profiles derived from 16S-amplicon and shotgun WGS: misaligned but isomorphic

**Fig. 2:**
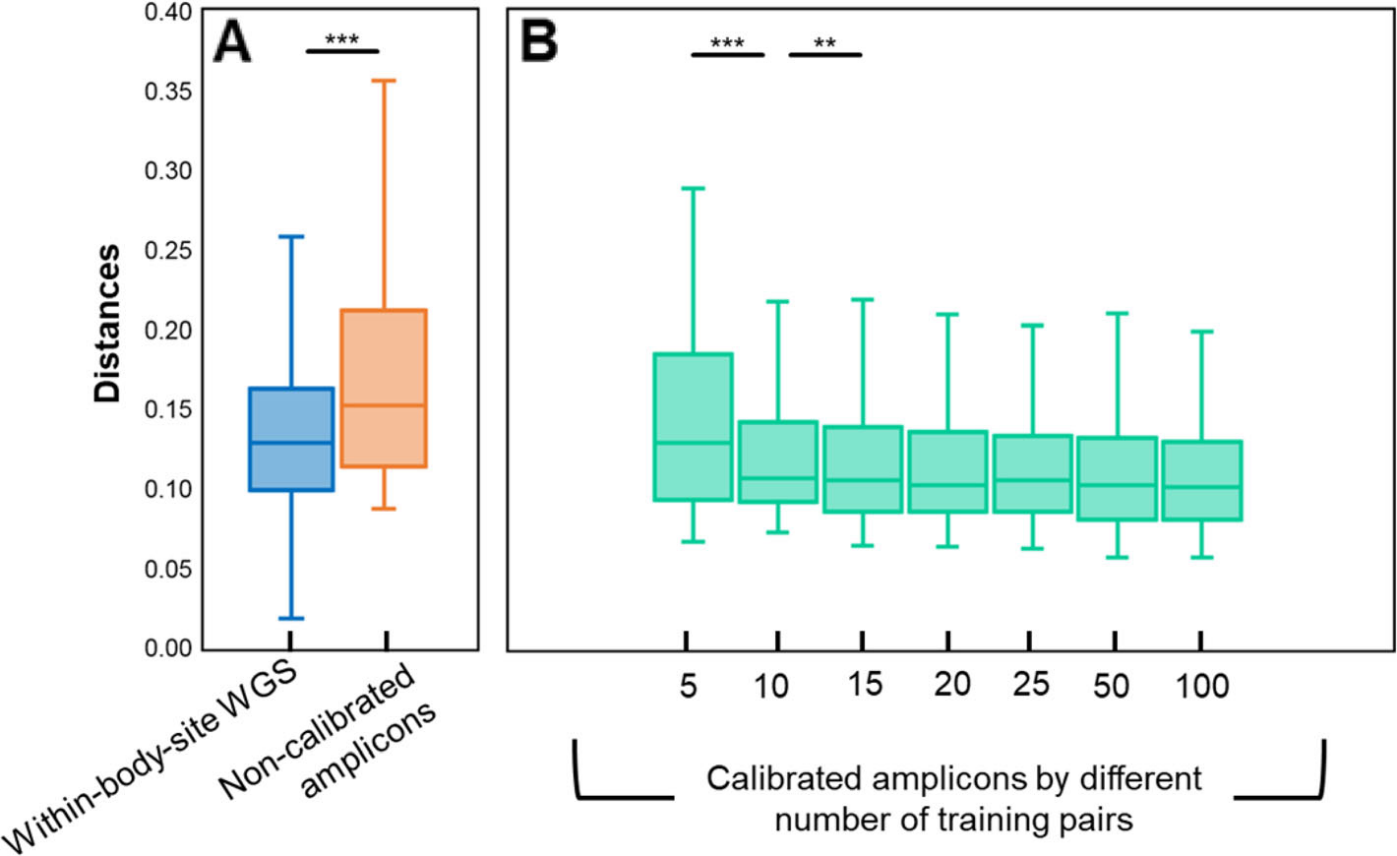
Meta-Apo significantly reduces the derivation of functional profile between WGS and amplicon sample pairs from Dataset 1. **a** The Bray-Curtis distances between WGS:16S amplicon pairs (without calibration, orange bar) are higher than those of the WGS within-body-site distance (distances among WGS samples of the same body site, blue bar). **b** The Bray-Curtis distances between calibrated amplicon samples and their paired WGS samples become stable when using only 15 training pairs, which is significantly lower than the within-group distances of WGS. Two panels share the x-axis. The *p*-values were calculated by two-tail Wilcox tests, ** denotes *p* < 0.05 and *** denotes *p* < 0.01

**Fig. 3:**
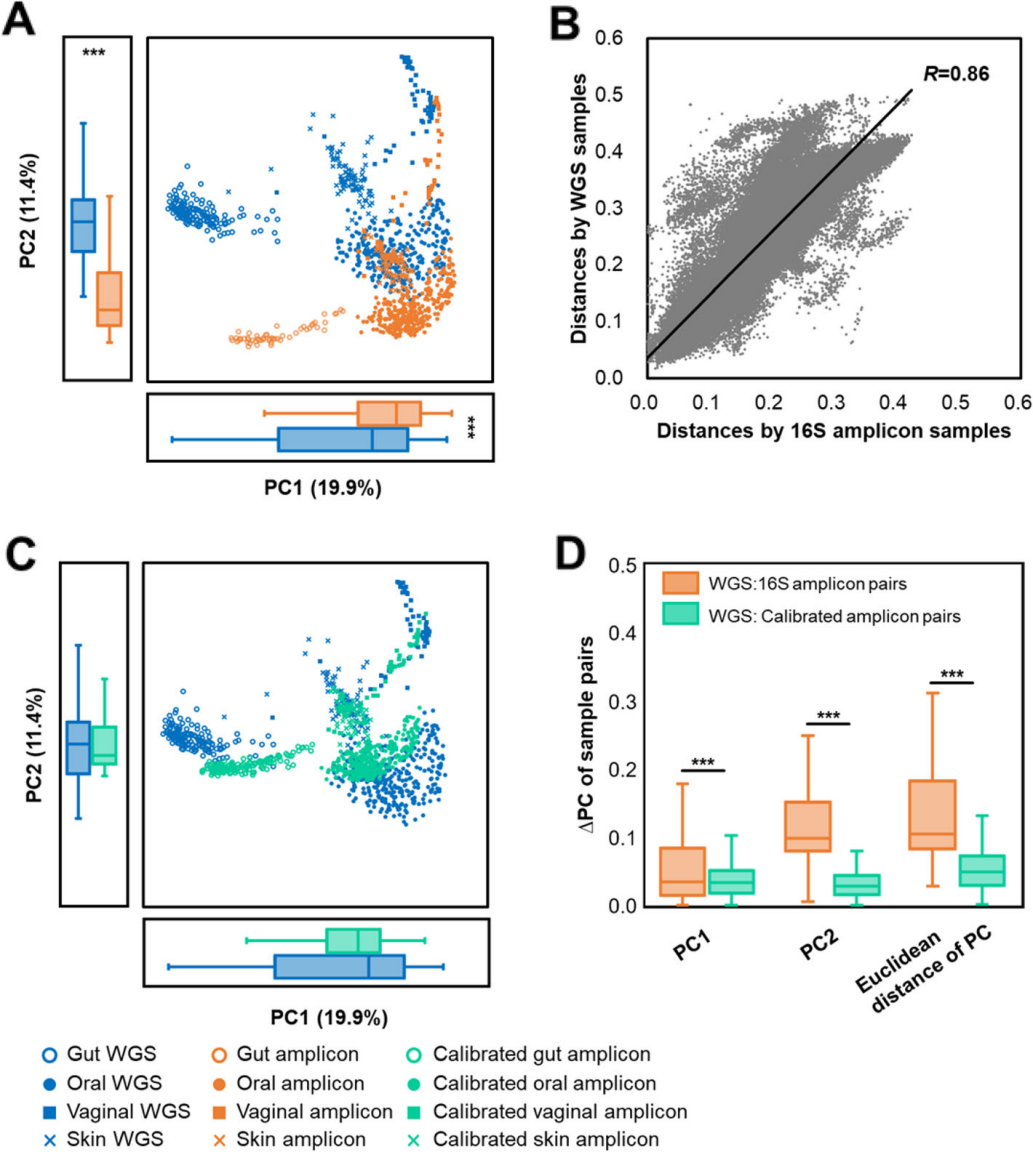
Functional beta diversity of the 622 WGS:16S-amplicon sample pairs from Dataset 1. **a** Overall functional patterns derived from the amplicon and WGS approaches are isomorphic but separate with significant differences on PC1 and PC2 distributions. **b** Bray-Curtis distances calculated by WGS and amplicons are strongly correlated (Pearson correlation *R* = 0.86, *p* < 0.01). **c** Meta-Apo aligns the predicted functional profiles derived from amplicon samples to those of WGS samples using 15 sample pairs for training, making the PC1 and PC2 of calibrated functional profiles are closer to WGS samples than the original, non-calibrated amplicon samples. **d** ∆PC of the WGS:16S amplicon pairs were significantly reduced. Principle coordinates were calculated by PCoA using the Bray-Curtis distances. The *p*-values were calculated by two-tail paired Wilcox tests, and *** denotes *p* < 0.01

To assess the degree of deviation in perceived microbiome function (annotated using KEGG Orthology [15]; KO) between the two sequencing strategies, we started by comparing the functional profiles of 622 paired human microbiomes (Dataset 1; four body sites: gut, skin, oral and vaginal; Table 1), each of which was sequenced via both shotgun WGS and V3-V5-region 16S rRNA amplicons. For WGS, the molecular functional profiles were derived via HUMAnN2 [17]. For 16S, the profiles were inferred using PICRUSt 2 [8] (Methods and Materials). By comparing the functional profiles derived from the two sequencing approaches, we found that the paired WGS:16S-amplicon distances were significantly higher than within-body-site distances of WGS (i.e., distances among WGS samples from the same body site; Fig. 2a; 0.166 ± 0.063 vs. 0.136 ± 0.056). Due to such a high degree of discrepancy between the two strategies, their beta-diversity exhibited very distinct patterns (Fig. 3a; PC1 two-tail paired Wilcox test *p* < 0.01; PC2 two-tail paired Wilcox test *p* < 0.01) and actually resulted in errors, e.g. the functional profiles of certain skin amplicons were incorrectly clustered as identical to those of oral WGS. On the other hand, pairwise distances derived from each of the two approaches were strongly correlated (Fig. 3b; Pearson correlation *R* = 0.86, *p* < 0.01), revealing a similar overall shape among the isomorphic beta-diversities (Fig. 3a; Monte-Carlo test *p* < 0.01). Therefore, functional profiles predicted from 16S amplicons (*K*_*16S*_) can be linked to those from WGS (*K*_*WGS*_) via eq. 1:

1 $$ {K}_{WGS}=f\left({K}_{16S}\right) $$

**Table 1:** The WGS and amplicon datasets used in this study

Dataset	# of WGS samples	# of amplicon samples	Amplicon type	Paired	Source study	Body site
**Dataset 1**	622	622	V3-V5 16S rRNA	Yes	HMP [2]	Gut, Oral, Skin and Vaginal
**Dataset 2**	295	295	V1-V3 16S rRNA	Yes	HMP [2]	Gut, Oral and Vaginal
**Dataset 3**	2354	5350	V3-V5 16S rRNA	No	HMP [2]	Gut, Oral, Skin and Vaginal
**Dataset 4**	2045	2186	V1-V3 16S rRNA	No	HMP [2]	Gut, Oral and Vaginal
**Dataset 5**	18	150	V1-V3 16S rRNA	Partially^a^	*ISME J.* 2014 [16]	Oral

^a^Only 18 WGS:16S-amplicon sample pairs

### Reduction of the deviation in functional profile between WGS and amplicon datasets by linear regression modeling

**Fig. 4:**
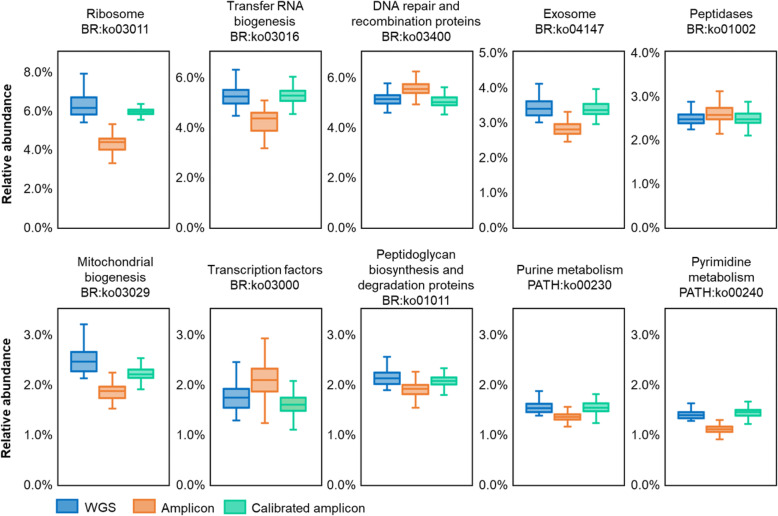
Comparison of the dominated functional profiles annotated by KEGG BRITE hierarchical level 3 classification.

Here we developed the Meta-Apo that exploited eq. 1 to reduce the deviation in functional profile between amplicon and WGS datasets. Meta-Apo consists of two steps: training and calibration. In the training step, Meta-Apo estimates the *f* of eq. 1 by a small number of WGS:16S-amplicon pairs using linear regression modeling. Then in the calibration step, by considering WGS results as the “golden standard”, Meta-Apo calibrates the predicted functional profiles of amplicon samples using model *f* (Methods and Materials for details). To quantitatively assess its performance, we randomly selected *N* = 5, 10, 15, 20, 50 and 100 WGS:16S-amplicon pairs from Dataset 1 as training, and used Meta-Apo to calibrate the other amplicon samples of this dataset (Methods and Materials for details). After such calibration, the paired WGS:16S-amplicon distances were significantly reduced, as compared to those derived from the same sets of un-calibrated samples (Fig. 2b; two-tail paired Wilcox test *p* < 0.01). Notably, such benefits by Meta-Apo-based calibration became stable when using model *f* that built from *N* = 15 training pairs, and did not change after adding more training pairs (up to 100; Fig. 2b). As a result, after the calibration (i.e., *N* = 15 training pairs), the paired WGS:16S-amplicon distances were significantly lower than the within-group distances of WGS samples (0.121 ± 0.055 vs. 0.136 ± 0.056). Principle Coordinate Analysis (PCoA) confirmed that Meta-Apo actually eliminated the overall functional-profile deviation between sample pairs produced by the two sequencing strategies (Fig. 3c, PC1 two-tail paired Wilcox test *p* = 0.30, PC2 two-tail paired Wilcox test *p* = 0.29; Fig. 3d). Further comparison on the dominated molecular function profiles annotated by KEGG BRITE hierarchical classification on all levels (level 3, Fig. 4; level 2, Fig. S1; level 1, Fig. S2) also suggested that the calibration of amplicons generated more consistent compositional relative abundances to the WGS than the original uncalibrated data (Fig. 4). Similarly, Meta-Apo was also effective for the V1-V3 region 16S rRNA sequences from Dataset 2 (Table 1), by accurately aligning amplicon- and WGS-derived functional patterns (Fig. S3 and Fig. S4).

### Calibration of predicted functions for 16S-amplicons on a large scale

**Fig. 5:**
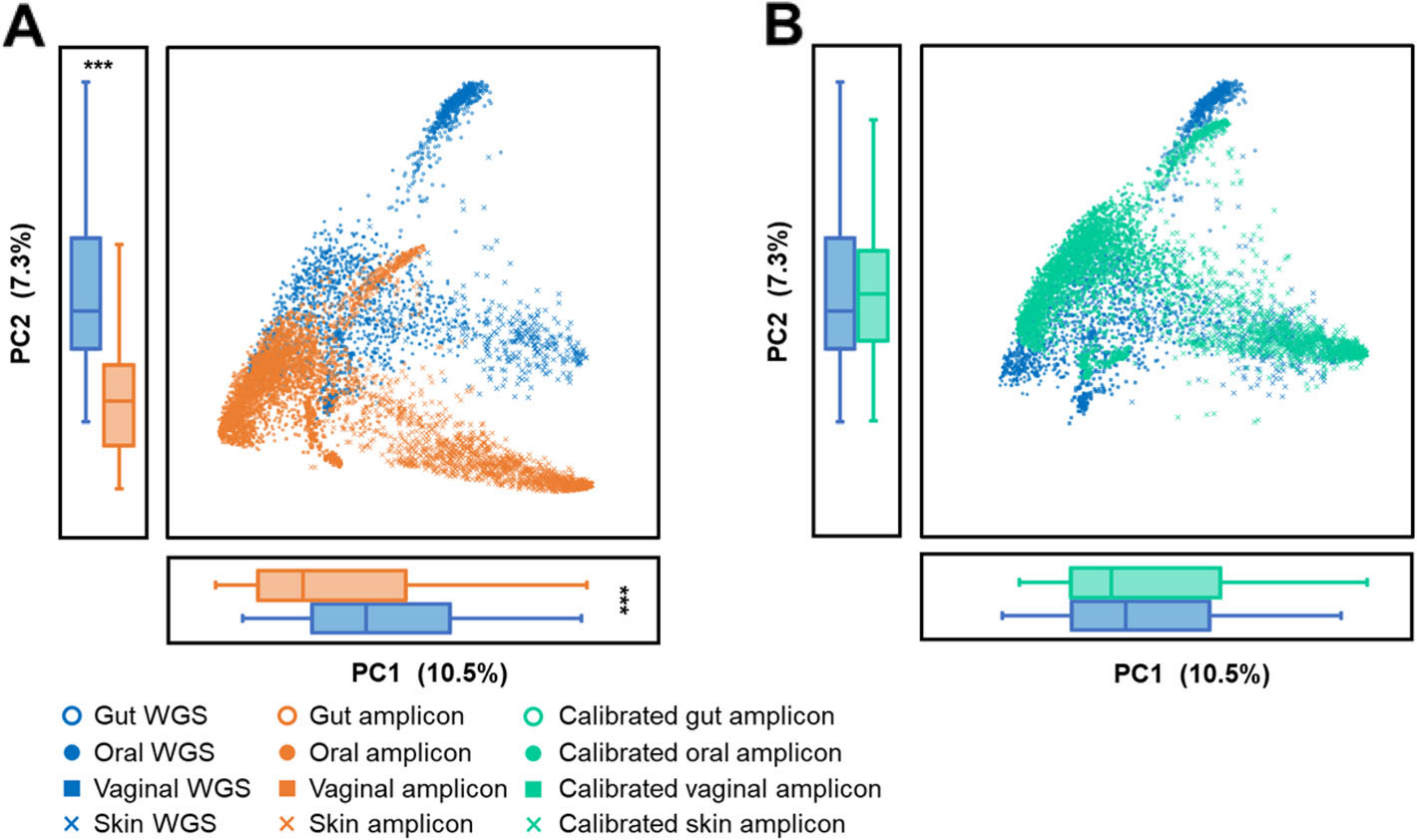
Functional beta diversity of the 2655 WGS samples and the 5350 amplicon samples from Dataset 3. **a** Functional patterns derived from the amplicon and WGS approaches are separate with significant differences on PC1 and PC2 distributions. **b** Meta-Apo aligns the predicted functional profiles of amplicon samples to those of the WGS samples using 15 sample pairs for training, making the PC1 and PC2 of calibrated functional profiles of amplicon samples are closer to WGS samples than the original, non-calibrated amplicon samples. Principle coordinates were calculated by PCoA using the Bray-Curtis distances. The *p*-values were calculated by two-tail Wilcox tests, and *** denotes *p* < 0.01

To evaluate the performance of such calibration for inferred functions on a large scale, we extended Meta-Apo to 5350 V3-V5 16S rRNA amplicon samples, and compared them to 2354 WGS samples (Dataset 3, collected from four body sites as Dataset 1, and sequences were processed using identical methods; Table 1). Although collected from the same body sites of the same healthy hosts and sequenced in the same study (Human Microbiome Project [2]; HMP), these WGS and amplicon samples were not paired, i.e., they are not sequenced from the same microbiome sample (in fact, such exactly paired data is usually not available at a large scale). On the other hand, the taxonomical composition in each of the body sites was internally consistent between WGS and amplicon (Fig. S5), i.e., regardless of the choice of sequencing strategy [18]. However, unlike the taxonomical diversity, the two strategies resulted in distinct functional patterns (Fig. 5a; PC1 two-tail Wilcox test *p* < 0.01; PC2 two-tail Wilcox test *p* < 0.01), e.g. gut amplicons were clustered with oral WGS, while oral samples were separated along the line of sequencing strategy. These observations, which contracted with previous findings that body site dominates the functional landscape of human microbiomes [2, 19], were likely due to the inaccuracy of 16S-amplicon-based functional prediction. We then calibrated the predictive functional profiles of all amplicon samples using Meta-Apo, via the same model constructed by 15 training WGS:16S-amplicon pairs of Dataset 1. Analysis of beta-diversity revealed that, after the calibration by Meta-Apo calibration, the deviation of functional profile between amplicon and WGS samples was greatly reduced (Fig. 5b; PC1 two-tail Wilcox test *p* = 0.20; PC2 two-tail Wilcox test *p* = 0.03).

Furthermore, to test its performance on 16S datasets of different priming regions, we applied Meta-Apo to 2186 V1-V3-region 16S-rRNA amplicon samples from Dataset 4 of HMP [2]; Table 1). Meta-Apo resulted in an equivalent degree of boost in the accuracy of amplicon-based functional profile reconstruction, using the model of WGS:16S-amplicon pairs of Dataset 2 (training pairs *N* = 15; Fig. S6). Therefore, Meta-Apo is generally applicable to the various priming regions of 16S rRNA genes.

### Calibration of functional profiles enables cross-platform comparison between WGS and amplicons and improves accuracy of disease-status classification

Shotgun WGS can provide more stable microbiome-based disease detection and classification across multiple studies than amplicons, due to their higher resolution and lower sequence amplification bias [13, 14]. However, shotgun WGS is not yet widely adopted for commercial or home microbiome test due to its higher cost in both experiment and analysis. Here using Dataset 5, we show that with a WGS-based disease classification method, the Meta-Apo-calibrated functional profiles inferred from 16S-amplicons can also obtain high classification accuracy, which is otherwise not possible for non-calibrated profiles. Dataset 5 contains 150 V1-V3-region 16S rRNA amplicon based human oral microbiomes with different disease status (healthy and gingivitis), in which 18 samples were also sequenced by shotgun sequencing [16] (Table 1**,** Table S1 and Methods and Materials). Therefore, we used the 18 WGS:16S-amplicon pairs to calibrate the inferred functional profiles of the other amplicon samples in this dataset, and evaluated the performance of Meta-Apo for cross-platform comparison and status identification.

**Fig. 6:**
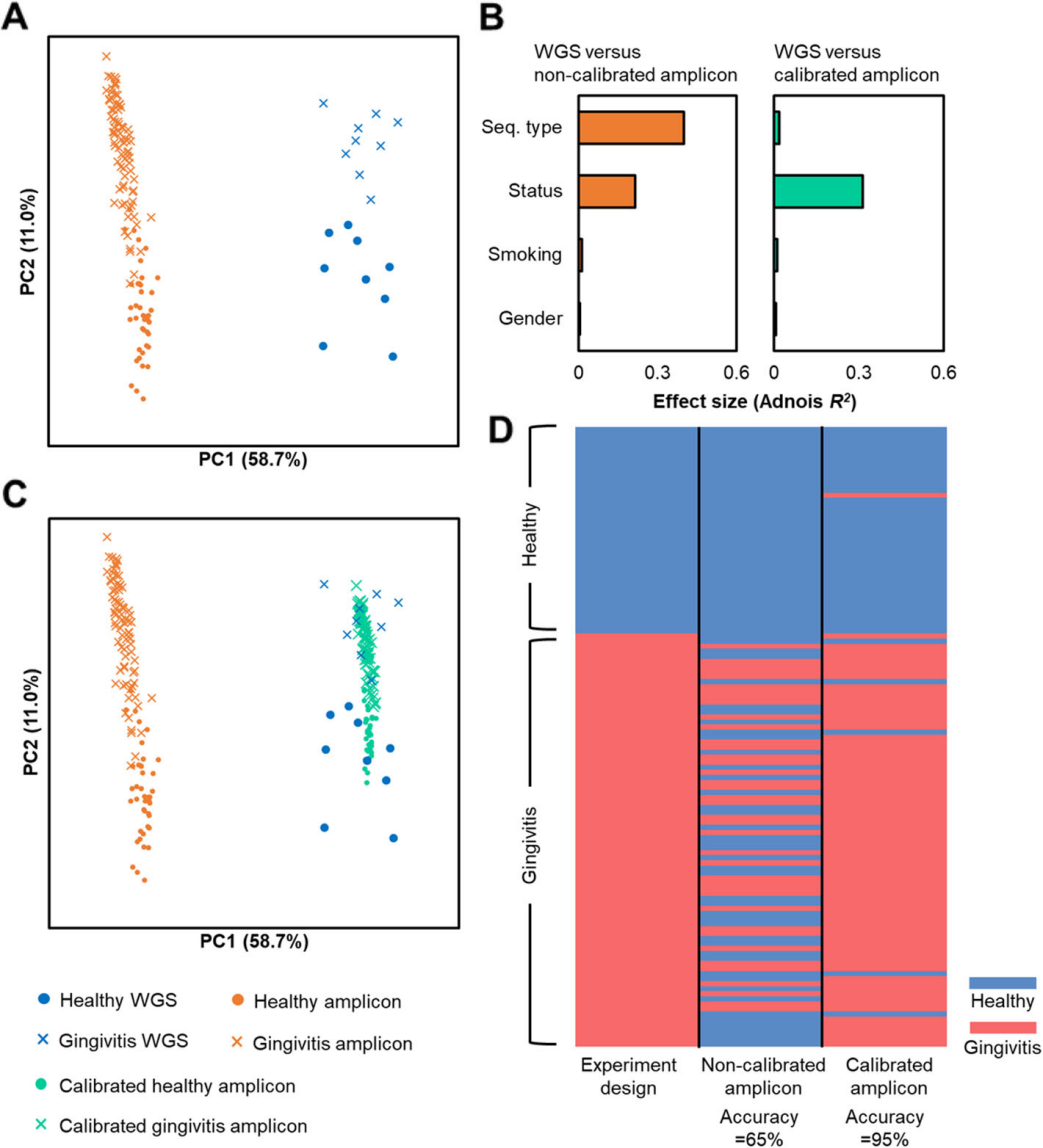
Cross-platform comparison of healthy and gingivitis oral microbiomes based on non-calibrated and calibrated functional profiles. **a** Functional patterns derived from the amplicon and WGS approaches are distinct, which suggests cross-platform comparison can be a significant challenge under such circumstances. **b** Comparing the effect size of the sampling factors by Adonis test. **c** Meta-Apo aligns the predicted functional profiles of amplicon samples to those of the WGS samples. **d** Healthy status classification of the original and the Meta-Apo-calibrated amplicon samples by MSE-based classification from WGS samples. Distances for Adonis test and PCoA were calculated using Bray-Curtis metrics

Although each of the two sequencing approaches was able to reveal the difference between healthy and disease microbiomes, the functional profiles of WGS and those predicted from amplicon samples exhibited a discrete pattern on the beta-diversity (Fig. 6a). In fact, the effect size (Adonis *R*^*2*^) of sequencing type exceeded that of disease status (Fig. 6b, left panel), underscoring the challenge of cross-platform comparison (i.e., between 16S-amplicon and WGS) under such circumstances. However, the calibration of Meta-Apo on amplicon samples diminished such deviation of reconstructed functional profile caused by the variation in sequencing strategy (Fig. 6c). As a result, the effect size of disease status dominated the sampling factors (Fig. 6b, right panel), suggesting the feasibility of microbiome-based disease classification. Therefore, Meta-Apo allows microbiome diagnosis that crosses the amplicon and WGS platforms.

To quantitatively assess the benefits of using Meta-Apo-calibrated 16S-amplicon-derived functional profiles for diagnosis, we performed a Microbiome Search Engine (MSE) based gingivitis classification [20, 21]. A database was first constructed by the functional features of 18 WGS samples, and then the disease status was predicted using the 123 original 16S and their corresponding Meta-Apo-calibrated amplicons, respectively (Methods and Materials for details; amplicon samples collected from the same hosts as the WGS were excluded to avoid prediction bias). Interestingly, the non-calibrated 16S-amplicon samples reported a low overall accuracy of 65.04% (F1-score = 0.6446) in cross-platform classification of disease status, mainly due to the insensitivity of detecting gingivitis subjects (recall = 0.4756; Fig. 6d). In contrast, after calibration by Meta-Apo, the accuracy of disease classification was raised to 95.12% (F1-score = 0.9570), while the sensitivity to the disease was also greatly improved (recall = 0.9390; details in Table S6). Therefore, for studies where both 16S amplicon and WGS types of data are available, Meta-Apo provides a strategy for cross-platform microbiome analysis that can significantly improve the performance of status classification.

### Meta-Apo calibration model for multiple categories: accuracy and comprehensiveness

Beta-diversity of microbial functions could be influenced by various factors (e.g. habitat, status, etc.). For example, human microbiomes of Dataset 1 were significantly differentiated by body-sites (Fig. 3a; Adonis test *p*-value < 0.01). To measure the sensitivity of Meta-Apo model to habitats, for skin samples in Dataset 1, we built additional two types of models by *N* = 15 training samples that *a*) all from skin and *b*) none from skin, respectively. Then we calculated the paired WGS:16S-amplicon distances in the same way as Fig. 2b (Methods and Materials for details). Result showed that distances were reduced by a model with only skin samples (Fig. S7A), suggesting the calibration accuracy of samples in a single category could be further improved by an appropriate category-specific model. On the other hand, such distances also enlarged that even worse than un-calibrated result when removing skin samples from training (Fig. S7A). This was mainly due the skin-free model lacked of adequate functional features that were abundant or unique in skin samples (Fig. S7B). Hence a model that covers all four body sites reduced the gap between sequencing types while kept the beta-diversity pattern among multiple habitats (Fig. 3c).

Furthermore, the category-specific model also exhibits shortage in applications of microbiome-based multi-category classification (e.g. disease detection), for the category information is always unknown (e.g. whether a sample is healthy or disease). Here, an arbitrary category-specific model may introduce bias to samples that belong other categories, leading to erroneous prediction results. For amplicon samples of Dataset 5, after calibration with a model that trained only by healthy WGS:16S-amplicon pairs, both healthy and disease samples were shifted to healthy WGS sample (Fig. S8A). Similarly, all samples were also recognized as unhealthy if the model only included disease pairs (Fig. S8B). In such case, a training set that includes both healthy and disease pairs is optimal. In summary, for calibration of microbiomes among multiple categories, if category information is definite (e.g. body-site), category-specific models will be ideal for each single category, while an integrated model that covers all categories also works well; otherwise (e.g. disease detection) an integrated model is suggested.

### Meta-Apo calibration model is experimental-protocol specific

Since the Meta-Apo builds a calibration model by solving *f* in eq. 1 using WGS:16S-amplicon pairs, it is important to note that the calibration model of Meta-Apo is not universal but specific to experimental protocols, i.e., amplicon sequences used in training and calibration should be produced via a consistent procedure. To test the effect of this issue, we calibrated the original oral amplicons of Dataset 5 using the calibration model constructed from the WGS:16S-amplicon pairs of Dataset 2. In both Dataset 2 and Dataset 5, WGS samples were sequenced by the Roche 454 platform, and the 16S rRNA sequences were amplified from the V1-V3 region and sequenced via the Illumina platform. However, the DNA was processed by different sampling and extraction protocols: Dataset 2 used the HMP 16S 454 protocol (http://hmpdacc.org), whereas Dataset 5 used the Larry Fernery’s protocol [22] with minor modifications (the two protocols mainly differ in primer design and DNA extraction method). As a result, after calibration by Meta-Apo, the effect size (Adonis *R*^*2*^) of sequencing type was still larger than that of the disease status, which is similar to the case of non-calibrated samples (Fig. S9A), and the beta-diversity pattern of calibrated amplicons was shifted towards an unexpected direction that is distinct from both the non-calibrated amplicons and the WGS samples (Fig. S9B). Therefore, the Meta-Apo calibration model is experimental-protocol specific, and transplantation of it among datasets generated from distinct experimental protocols (such as those related to primer design and DNA extraction) should take caution.

## Conclusion and discussion

The rapid expansion of microbiome research has driven large-scale analyses of microbiome, from both taxonomical and functional perspectives [20]. At present, due to their much lower experimental and computation costs, 16S-amplicon-based samples still outnumber WGS-based samples by a factor of 100. In addition, in circumstances where biomass is not adequate for shotgun WGS, 16S-amplicon based methods are still more practical. Therefore, the ability to accurately and reliably reconstruct microbiome function based on 16S-amplicon datasets would greatly accelerate large-scale microbiome data mining and thus is highly desirable. Meta-Apo fills such a niche, by calibrating the functional profiles for a large number (e.g. over 5000) of 16S-amplicon samples via only a much smaller number (e.g., 15–20 human microbiomes) of WGS:16S-amplicon paired samples for training. Adopting this strategy and tool, large-scale, function-oriented microbiome sequencing projects can probably benefit from the lower cost of 16S-amplicon strategy, yet without sacrificing the higher precision in functional reconstruction of WGS strategy.

Notably, the accuracy of 16S rRNA-based functional reconstruction is also dependent on the resolution of taxonomy profiling [23] and the number of reference genomes available [7]. At present, the number of complete microbial genomes for 16S-based functional profile prediction is over 20,000 [24], and they are mainly from human microbiota (e.g. reference database of PICRUSt 2). This is still one to two orders of magnitudes lower than the number of known 16S rRNA genes [25] (e.g. reference full-length 16S rRNA already number over 2 millions), therefore the calibration of 16S-based functional profiles for environmental microbiomes, in which complete reference genomes are much more sparse, can be more difficult. On the other side, currently the calibration of amplicon-based functional profiling for environmental microbiomes is also limited by the lack of appropriate WGS:16S-amplicon sample pairs. However, technology development in large-scale cultivation-based [26, 27] or single-cell-based sequencing [28] are rapidly elevating the number of novel microbial genomes (and their associated 16S rRNA genes). Therefore, with new tools such as Meta-Apo, amplicon-based sequencing and analysis strategy should continue to contribute to functional interrogation of microbiota, for both historical and emerging microbiome projects.

## Methods and materials

Meta-Apo takes the functional profiles of a small number of WGS:16S-amplicon sample pairs as training, and outputs the calibrated functional profiles of large-scale amplicon samples. It consists of two steps: training and calibration. In the training step, Meta-Apo estimates the *f* of eq. 1 by a small number of WGS:16S-amplicon pairs using linear regression modeling. Then in the calibration step, considering WGS results as the “golden standard”, Meta-Apo calibrates the predicted functional profiles of amplicon samples using model *f.*

### The training step of meta-Apo

Basically, the functional profile of a single microbiome *K*_*microbiome*_ consists of a series of molecular functions (e.g. KEGG Orthology) and their relative abundance as follows:

2 $$ {K}_{microbiome}=\left\{{k}_{function\ 1},{k}_{function\ 2},\dots, {k}_{function\ i}\right\} $$

in which *k*_*function i*_ represents the relative abundance of a molecular function. Based on the isomorphism (Fig. 3a; Monte-Carlo test *p* < 0.01) and strong linear correlation (Fig. 3b; Pearson correlation *R* = 0.86, *p* < 0.01) of functional profiles between WGS and amplicons, for each function, we can link their relative abundance values between the two approaches by further transforming eq. 1 as

3 $$ {k}_{WGS}=f\left({k}_{16S}\right)={\theta}_0{k}_{16S}+{\theta}_1 $$

In eq. 3, Meta-Apo solves the mapping model *f* by linear regression algorithm using function profiles of *N* (e.g. *N* = 15) exactly paired WGS:16S-amplicon samples as training. Specifically, Meta-Apo calculates the two parameters of *θ*_0_ and *θ*_1_ in eq. 3 for each function that fits *k*_*16s*_ to *k*_*WGS*_ by minimizing their total square error in eq. 4:

4 $$ E=\frac{1}{2}{\sum}_{i=1}^N{\left(f\left({k}_{16S}\right)-{k}_{WGS}\right)}^2 $$

Thus, in the training step, Meta-Apo calculates the parameters *θ*_1_ and *θ*_0_ by the Least Square Method (LSM) solution described in eq. 5:

5 $$ {\displaystyle \begin{array}{c}{\theta}_0=\frac{N\ast {\sum}_{i=1}^N{k}_{16s}\ast {k}_{WGS}-{\sum}_{i=1}^N{k}_{16s}\ast {\sum}_{i=1}^N{k}_{WGS}}{N\ast {\sum}_{i=1}^N{k_{16s}}^2-{\left({\sum}_{i=1}^N{k}_{16S}\right)}^2}\\ {}{\theta}_1=\frac{\sum_{i=1}^N{k_{16s}}^2\ast {\sum}_{i=1}^N{k}_{WGS}-{\sum}_{i=1}^N\left({k}_{16s}\ast {k}_{WGS}\right)\ast {\sum}_{i=1}^N{k}_{16s}}{N\ast {\sum}_{i=1}^N{k_{16s}}^2-{\left({\sum}_{i=1}^N{k}_{16S}\right)}^2}\end{array}} $$

### The calibration step of meta-Apo

With the optimal mapping model *f* for each molecular function derived from data training, Meta-Apo estimates the expected relative abundance of each input molecular function that was inferred from 16S rRNA amplicon sequences by eq. 6

6 $$ {k}_{expected}={\theta}_1{k}_{16S}+{\theta}_0\approx {k}_{WGS} $$

Since the mapping model has been optimized to minimize the difference between expected and “real” function abundance derived from WGS data, Meta-Apo calibrates the predicted functional profiles of amplicon samples to the WGS-level resolution.

### Datasets and profiling

In this work we prepared five human microbiome datasets from two studies (Table 1) to evaluate the performance of Meta-Apo. Samples in Dataset 1, 2, 3 and 4 were produced by HMP phase I [2] that collected from four body sites (gut, oral, skin and vaginal) of healthy hosts (downloaded from Data Analysis and Coordination Center of HMP at https://www.hmpdacc.org/hmp/, details available in Table S1, S2, S3 and S4). Dataset 1 contains 622 paired WGS:16S-amplicon microbiomes that each sample was sequenced by both WGS and V3-V5 region 16S rRNA amplicon sequencing. Dataset 2 contains 295 sample pairs of WGS and V1-V3 region 16S rRNA amplicon samples. Dataset 3 contains 2354 WGS samples and 5350 V3-V5 16S rRNA amplicon samples. Dataset 4 contains 2045 WGS samples and 2186 V1-V3 16S rRNA amplicon samples. Samples in Dataset 3 and Dataset 4 are not paired but collected from the same body sites of healthy hosts and sequenced by the same study along with consistent protocol. Dataset 3 and Dataset 4 share identical WGS samples, however, due to the lack of WGS and V1-V3 amplicon pairs from skin in Dataset 2 for the training purpose, we also removed the skin samples from Dataset 4 for the testing (the sample number of Dataset 4 in Table 1 was after such removal). Dataset 5 was produced by Huang, et al., *ISME J*. 2014 [16], which was collected from oral microbial communities of healthy and gingivitis hosts. It contains 150 V1-V3-region 16S rRNA amplicon oral microbiomes (50 healthy and 100 gingivitis), in which 18 samples (9 healthy and 9 gingivitis) were also sequenced by shotgun sequencing (details available in Table S5).

For all WGS samples, functional profiles were directly analyzed by HUMAnN2 [17] and annotated with KEGG Orology (KO), and taxonomical compositions on Genus level were analyzed by MetaPhlAn 2 [29]. For the 16S rRNA amplicon sequences, Operational Taxonomy Units (OTUs) were picked and annotated against Green-Genes (version 13–8) [30] database with cutoff similarity of 97% by Parallel-META 3 [31] and taxonomical relative abundances on Genus level were calibrated by 16S rRNA copy number from IMG/M database [32], then the KO functional profiles were inferred by PICRUSt 2 [8].

### Random sample selection for construction of calibration training model

To test the performance of Meta-Apo, we randomly selected *N* = 5, 10, 15, 20, 50 and 100 WGS:16S-amplicon pairs from Dataset 1 as training, and used Meta-Apo to calibrate the other amplicon samples of this dataset. The procedure of each *N* that includes sample selection, model training and calibration was repeated for 10 times, and iterations on each *N* were performed respectively. In addition, we also ensured that the *N* training samples covered all body sites of Dataset 1 (Table 1; Gut, Oral, Skin and Vaginal). After calibration, the paired WGS:16S-amplicon distances of each *N* (Fig. 2b) were calculated by the mean values of the 10 repeats. We then randomly chose a calibration model from the 10 repeats of *N* = 15 for further PCoA (Fig. 3c and d) and pathway analysis (Fig. 4) of Dataset 1, as well as the calibration and PCoA of Dataset 3 (Fig. 5b). Results of Dataset 2 (Fig. S1 and Fig. S2C) and Dataset 4 (Fig. S4) were produced in the same way.

### Cross-platform disease classification using microbiome functional profiles

For Dataset 5, we constructed the MSE [20] database with KO profiles analyzed from 18 WGS samples (9 healthy and 9 gingivitis; Table S5). Then each of the original amplicon samples and the calibrated amplicon samples was searched against this database using KO profiles for status classification [21]. Amplicon samples collected from the same hosts as the WGS were excluded from this testing to avoid the prediction and statistical bias. The classification results were evaluated from the follow aspects:

- accuracy = (true positive + true negative) / (# of all test samples)
- F1-score = (2 * recall * precision) / (recall + precision), in which
  recall = (true positive) / (true positive + false negative)
  precision = (true positive) / (true positive + false positive)

### Software packages for PCoA and statistics

The Principle Coordinate Analysis was performed by the “vegan” [33] package in R [34]. The two-tail Wilcox tests were performed by the “wilcox.test” function in R with “two.sided” parameter and 95% confidence level (conf.level = 0.95). The Monte-Carlo tests were performed by the “ade4” [35] package in R with 10,000 times permutation.

## Supplementary Information

**Additional file 1: Figure S1.** Comparison of the dominated functional profiles annotated by KEGG BRITE hierarchical level 2 classification. **Figure S2.** Comparison of the dominated functional profiles annotated by KEGG BRITE hierarchical level 1 classification. **Figure S3.** Meta-Apo significantly reduces the derivation of functional profile between amplicon and WGS datasets from Dataset 2. **Figure S4.** Functional beta diversity of the 295 WGS-amplicon sample pairs of Dataset 2. **Figure S5.** The 2655 WGS samples and the 5350 amplicon samples from Dataset 3 have consistent overall taxonomical patterns at the Genus level. **Figure S6.** Functional beta diversity of the 2045 WGS samples and the 2186 V1-V3 region amplicon samples from Dataset 4. **Figure S7.** Calibration of skin amplicons using different habitat models. **Figure S8.** Calibration of amplicons for disease detection using status-specific models. **Figure S9.** Calibration of amplicons using training samples that produced under inconsistent experiment protocols.

**Additional file 2: Table S1.** Detailed information of Dataset 1. **Table S2.** Detailed information of Dataset 2. **Table S3.** Detailed information of Dataset 3. **Table S4.** Detailed information of Dataset 4. **Table S5.** Detailed information of Dataset 5. **Table S6.** Healthy status classification result of original and calibrated amplicon samples by WGS-based classification.

## Data Availability

An optimized C++ implementation of Meta-Apo is available on GitHub (https://github.com/qibebt-bioinfo/meta-apo) under a GNU GPL license. It takes the functional profiles of a few paired WGS:16S-amplicon samples as training, and outputs the calibrated functional profiles for a much larger number of 16S-amplicon samples. The summary of all datasets used in this work are available from the papers listed in Table 1, and the accession IDs are listed in Table S1, S2, S3, S4 and S5. The functional profile tables and analysis scripts of all datasets have been uploaded to the Meta-Apo GitHub page. All other relevant data is available upon request.

## Abbreviations

Meta-Apo: Metagenomic Apochromat
WGS: Whole Genome Sequencing
PCR: Polymerase Chain Reaction
KEGG: Kyoto Encyclopedia of Genes and Genomes
KO: KEGG Orthology
PCoA: Principle Coordinate Analysis
HMP: Human Microbiome Project
MSE: Microbiome Search Engine
OTU: Operational Taxonomy Unit

## References

[1] Integrative HMPRNC (2019). The integrative human microbiome project. Nature.

[2] Human Microbiome Project C (2012). Structure, function and diversity of the healthy human microbiome. Nature.

[3] Knight R, Vrbanac A, Taylor BC, Aksenov A, Callewaert C, Debelius J (2018). Best practices for analysing microbiomes. Nat Rev Microbiol.

[4] Abubucker S, Segata N, Goll J, Schubert AM, Izard J, Cantarel BL (2012). Metabolic reconstruction for metagenomic data and its application to the human microbiome. PLoS Comput Biol.

[5] Morgan XC, Huttenhower C (2012). Chapter 12: human microbiome analysis. PLoS Comput Biol.

[6] Su X, Jing G, Zhang Y, Wu S (2020). Method development for cross-study microbiome data mining: challenges and opportunities. Comput Struct Biotechnol J.

[7] Langille MG, Zaneveld J, Caporaso JG, McDonald D, Knights D, Reyes JA (2013). Predictive functional profiling of microbial communities using 16S rRNA marker gene sequences. Nat Biotechnol.

[8] Douglas GM, Maffei VJ, Zaneveld JR, Yurgel SN, Brown JR, Taylor CM (2020). PICRUSt2 for prediction of metagenome functions. Nat Biotechnol.

[9] Asshauer KP, Wemheuer B, Daniel R, Meinicke P (2015). Tax4Fun: predicting functional profiles from metagenomic 16S rRNA data. Bioinformatics.

[10] Jun SR, Robeson MS, Hauser LJ, Schadt CW, Gorin AA (2015). PanFP: pangenome-based functional profiles for microbial communities. BMC Res Notes.

[11] Walker AW, Martin JC, Scott P, Parkhill J, Flint HJ, Scott KP. 16S rRNA gene-based profiling of the human infant gut microbiota is strongly influenced by sample processing and PCR primer choice. Microbiome. 2015;3. 10.1186/s40168-015-0087-4 PMC4482049 26120470

[12] Bonnet R, Suau A, Dore J, Gibson GR, Collins MD (2002). Differences in rDNA libraries of faecal bacteria derived from 10-and 25-cycle PCRs. Int J Syst Evol Microbiol.

[13] Wirbel J, Pyl PT, Kartal E, Zych K, Kashani A, Milanese A (2019). Meta-analysis of fecal metagenomes reveals global microbial signatures that are specific for colorectal cancer. Nat Med.

[14] Jackson MA, Verdi S, Maxan ME, Shin CM, Zierer J, Bowyer RCE (2018). Gut microbiota associations with common diseases and prescription medications in a population-based cohort. Nat Commun.

[15] Minoru K, Susumu G, Yoko S, Miho F, TJNAR M. KEGG for integration and interpretation of large-scale molecular data sets. 2012;40(D1):D109–14. 10.1093/nar/gkr988 PMC3245020 22080510

[16] Huang S, Li R, Zeng X, He T, Zhao H, Chang A (2014). Predictive modeling of gingivitis severity and susceptibility via oral microbiota. ISME J.

[17] Franzosa EA, McIver LJ, Rahnavard G, Thompson LR, Schirmer M, Weingart G (2018). Species-level functional profiling of metagenomes and metatranscriptomes. Nat Methods.

[18] Rausch P, Ruhlemann M, Hermes BM, Doms S, Dagan T, Dierking K (2019). Comparative analysis of amplicon and metagenomic sequencing methods reveals key features in the evolution of animal metaorganisms. Microbiome.

[19] Turnbaugh PJ, Hamady M, Yatsunenko T, Cantarel BL, Duncan A, Ley RE (2009). A core gut microbiome in obese and lean twins. Nature.

[20] Su X, Jing G, McDonald D, Wang H, Wang Z, Gonzalez A (2018). Identifying and predicting novelty in microbiome studies. MBio.

[21] Su X, Jing G, Sun Z, Liu L, Xu Z, McDonald D (2020). Multiple-disease detection and classification across cohorts via microbiome search. mSystems.

[22] Ravel J, Gajer P, Abdo Z, Schneider GM, Koenig SS, McCulle SL (2011). Vaginal microbiome of reproductive-age women. Proc Natl Acad Sci U S A.

[23] Yarza P, Yilmaz P, Pruesse E, Glockner FO, Ludwig W, Schleifer KH (2014). Uniting the classification of cultured and uncultured bacteria and archaea using 16S rRNA gene sequences. Nat Rev Microbiol.

[24] Haft DH, DiCuccio M, Badretdin A, Brover V, Chetvernin V, O'Neill K (2018). RefSeq: an update on prokaryotic genome annotation and curation. Nucleic Acids Res.

[25] Quast C, Pruesse E, Yilmaz P, Gerken J, Schweer T, Yarza P (2013). The SILVA ribosomal RNA gene database project: improved data processing and web-based tools. Nucleic Acids Res.

[26] Zou Y, Xue W, Luo G, Deng Z, Qin P, Guo R (2019). 1,520 reference genomes from cultivated human gut bacteria enable functional microbiome analyses. Nat Biotechnol.

[27] Forster SC, Kumar N, Anonye BO, Almeida A, Viciani E, Stares MD (2019). A human gut bacterial genome and culture collection for improved metagenomic analyses. Nat Biotechnol.

[28] Xu J, Ma B, Su XQ, Huang S, Xu X, Zhou XD (2017). Emerging trends for microbiome analysis: from single-cell functional imaging to microbiome big data. Engineering.

[29] Truong DT, Franzosa EA, Tickle TL, Scholz M, Weingart G, Pasolli E (2015). MetaPhlAn2 for enhanced metagenomic taxonomic profiling. Nat Methods.

[30] McDonald D, Price MN, Goodrich J, Nawrocki EP, DeSantis TZ, Probst A (2012). An improved Greengenes taxonomy with explicit ranks for ecological and evolutionary analyses of bacteria and archaea. ISME J.

[31] Jing G, Sun Z, Wang H, Gong Y, Huang S, Ning K (2017). Parallel-META 3: comprehensive taxonomical and functional analysis platform for efficient comparison of microbial communities. Sci Rep-Uk.

[32] Chen IA, Chu K, Palaniappan K, Pillay M, Ratner A, Huang J (2019). IMG/M v.5.0: an integrated data management and comparative analysis system for microbial genomes and microbiomes. Nucleic Acids Res.

[33] Dixon P (2003). VEGAN, a package of R functions for community ecology. J Veg Sci.

[34] R-Core-Team (ed.) (2013). R: a language and environment for statistical computing.

[35] Dray S, Dufour AB (2007). The ade4 package: implementing the duality diagram for ecologists. J Stat Softw.

