# Supplementary material for: Meta-Apo improves accuracy of 16S-amplicon-based prediction of microbiome function
Source: BMC Genomics. 2021 Jan 6;22:9. doi: 10.1186/s12864-020-07307-1 (PMC7788972; doi:10.1186/s12864-020-07307-1)
Supplement: Supplementary file 1 — Additional file 1: Figure S1. Comparison of the dominated functional profiles annotated by KEGG BRITE hierarchical level 2 classification. Figure S2. Comparison of the dominated functional profiles annotated by KEGG BRITE hierarchical level 1 classification. Figure S3. Meta-Apo significantly reduces the derivation of functional profile between amplicon and WGS datasets from Dataset 2. Figure S4. Functional beta diversity of the 295 WGS-amplicon sample pairs of Dataset 2. Figure S5. The 2655 WGS samples and the 5350 amplicon samples from Dataset 3 have consistent overall taxonomical patterns at the Genus level. Figure S6. Functional beta diversity of the 2045 WGS samples and the 2186 V1-V3 region amplicon samples from Dataset 4. Figure S7. Calibration of skin amplicons using different habitat models. Figure S8. Calibration of amplicons for disease detection using status-specific models. Figure S9. Calibration of amplicons using training samples that produced under inconsistent experiment protocols. [file 12864_2020_7307_MOESM1_ESM.docx]

**Supplemental figures**


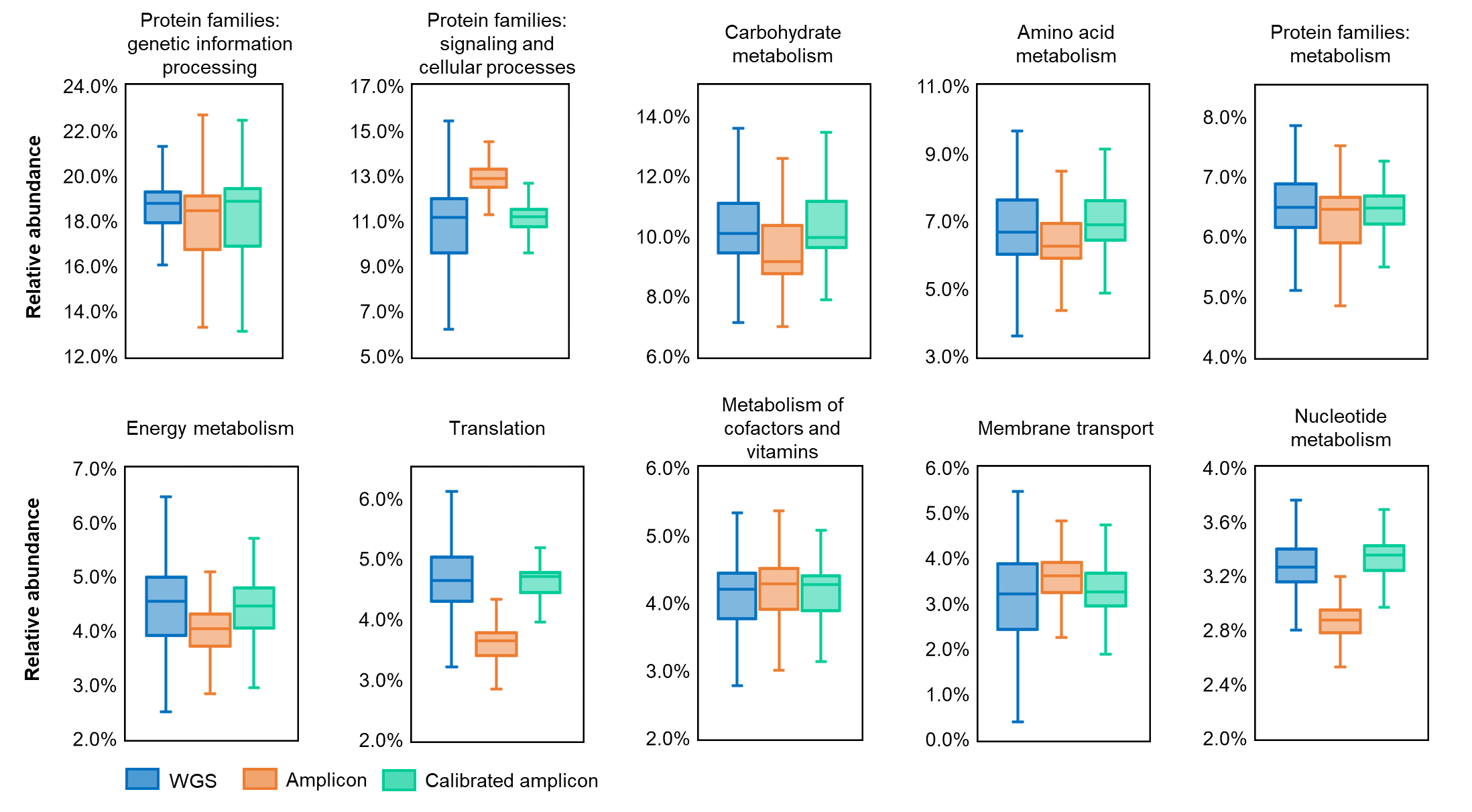


**Fig. S1. Comparison of the dominated functional profiles annotated by KEGG BRITE hierarchical level 2 classification.**

**
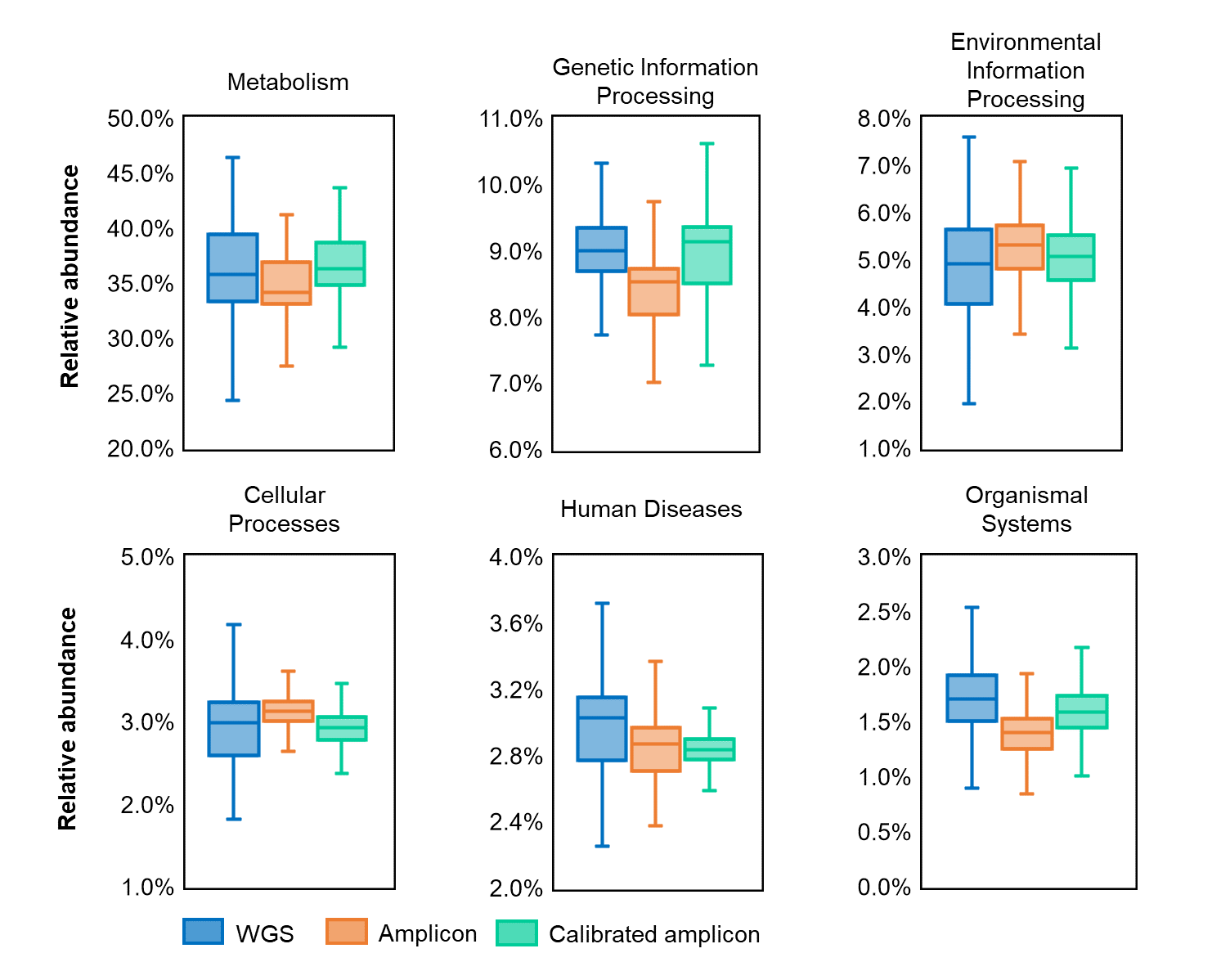
**

**Fig. S2. Comparison of the dominated functional profiles annotated by KEGG BRITE hierarchical level 1 classification.**

**
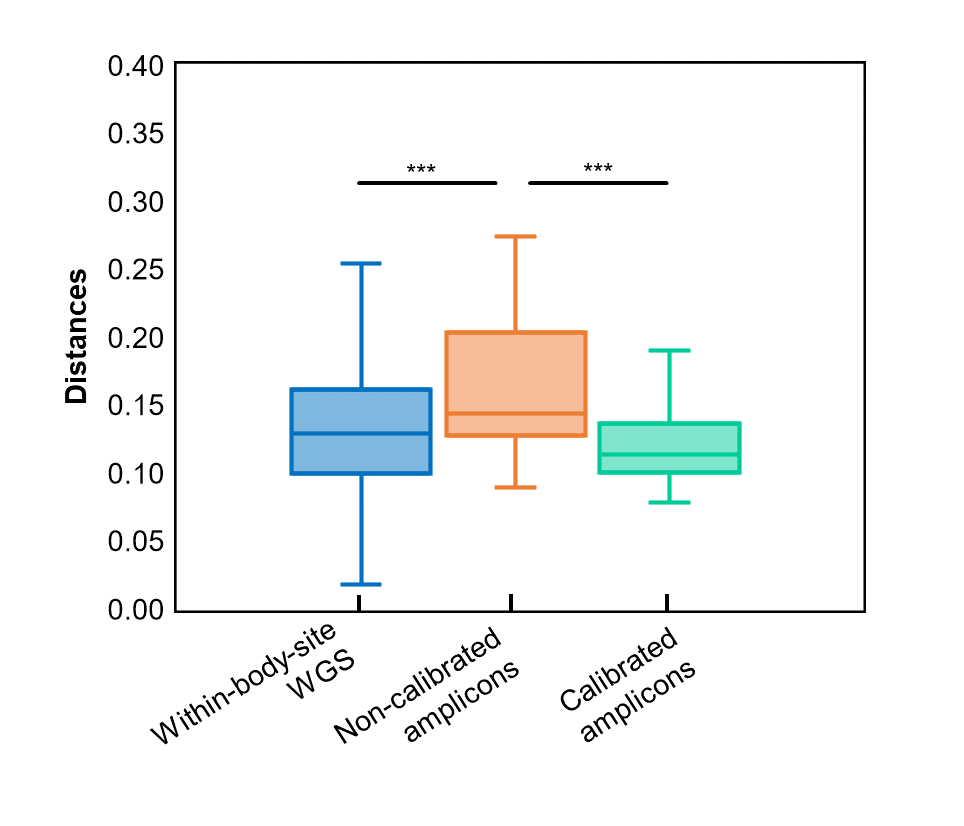
**

**Fig. S3. Meta-Apo significantly reduces the derivation of functional profile between amplicon and WGS datasets from Dataset 2.** The Bray-Curtis distances between WGS and paired amplicon samples (without calibration, orange bar) are higher than those of the WGS within-body-site distance (distances among WGS samples of the same body site, blue bar; 0164 ± 0.044 vs. 0.135 ± 0.050). After calibration using only 15 training pairs, the Bray-Curtis distances between amplicon samples and their paired WGS samples is significantly reduced and thus lower than the within-group distances of WGS (green bar, 0.124 ± 0.035 vs. 0.135 ± 0.050). The *p*-values were calculated by two-tail Wilcox tests, and *** denotes *p*<0.01.

**
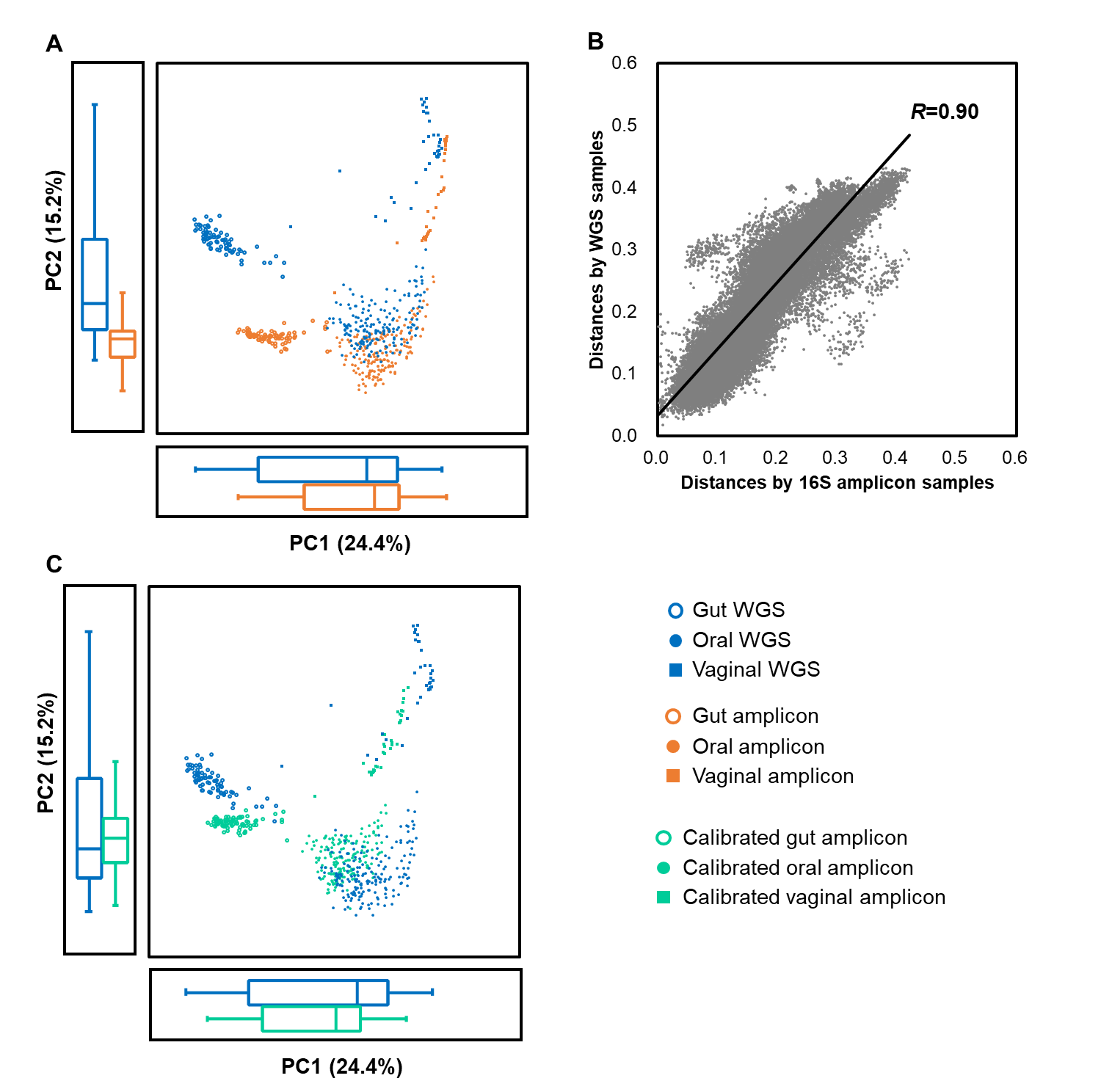
**

**Fig. S4. Functional beta diversity of the 295 WGS-amplicon sample pairs of Dataset 2.** (**A**) Overall functional patterns derived from the amplicon and WGS approaches are isomorphic (Monte-Carlo test *p*-value < 0.01) but separate. (**B**) Distances calculated by WGS and amplicons are strongly correlated (Pearson correlation *R*=0.90, *p*-value < 0.01). (**C**) Meta-Apo aligns the predicted functional-gene profiles derived from amplicon samples to those of WGS samples using 15 sample pairs for training. Principle coordinates are calculated by PCoA using the Bray-Curtis distances.


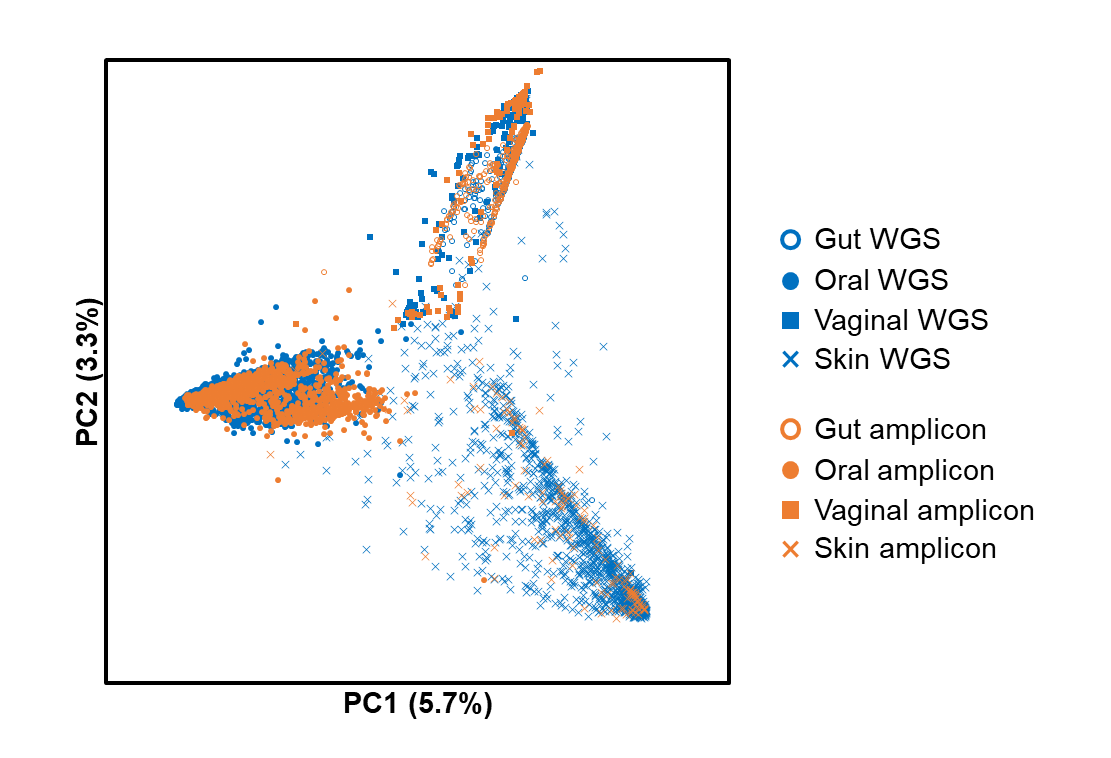


**Fig. S5. The 2,655 WGS samples and the 5,350 amplicon samples from Dataset 3 have consistent overall taxonomical patterns at the Genus level.** Principle coordinates were calculated by PCoA using the Bray-Curtis distances of genus-level profiles.


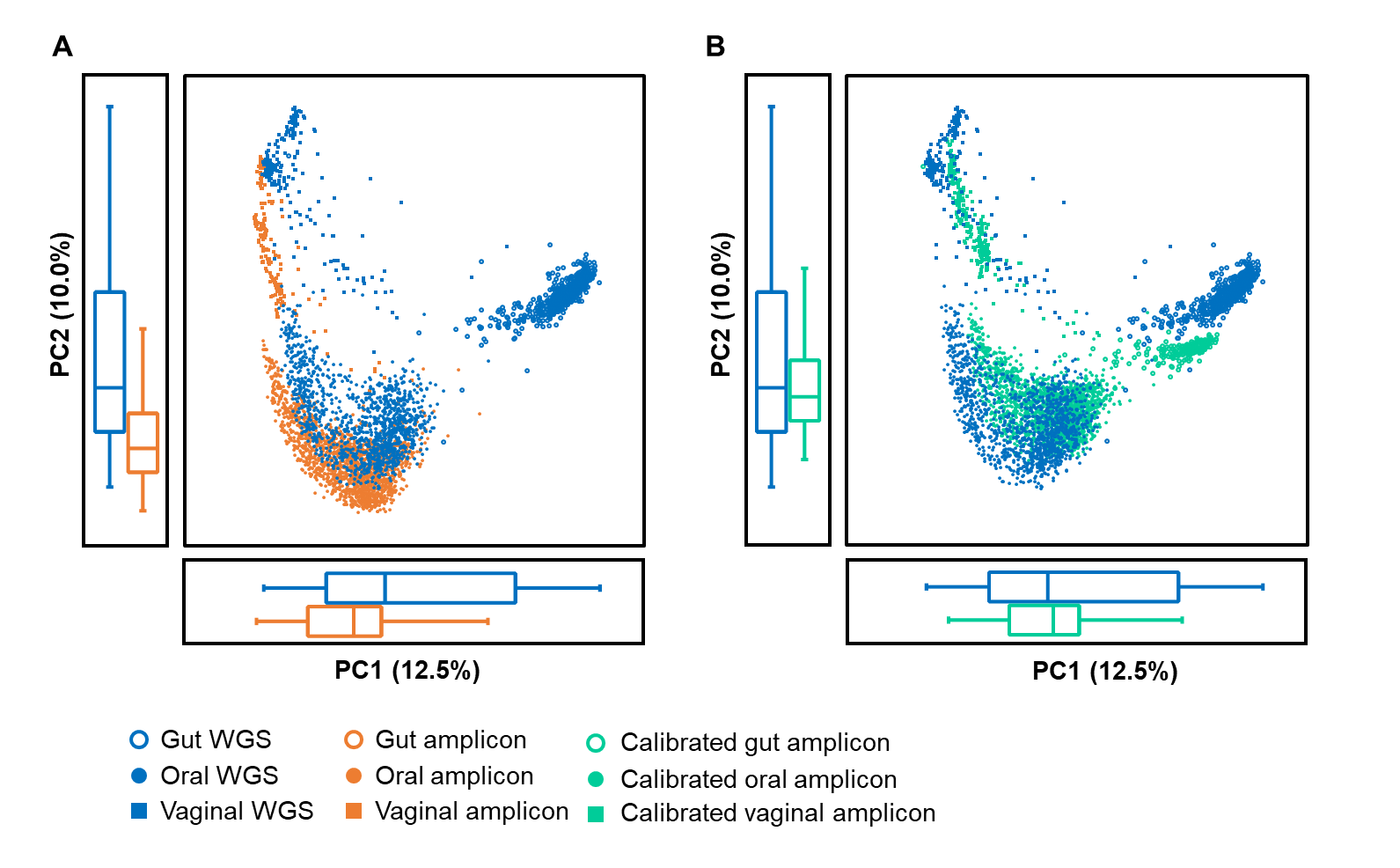


**Fig. S6. Functional beta diversity of the 2,045 WGS samples and the 2,186 V1-V3 region amplicon samples from Dataset 4.** (**A**) Overall functional patterns derived from the two strategies are separate. (**B**) Meta-Apo aligns the predicted functional-gene profiles of amplicon samples to those of the WGS samples using 15 sample pairs for training. Principle coordinates are calculated by PCoA using the Bray-Curtis distances.

**
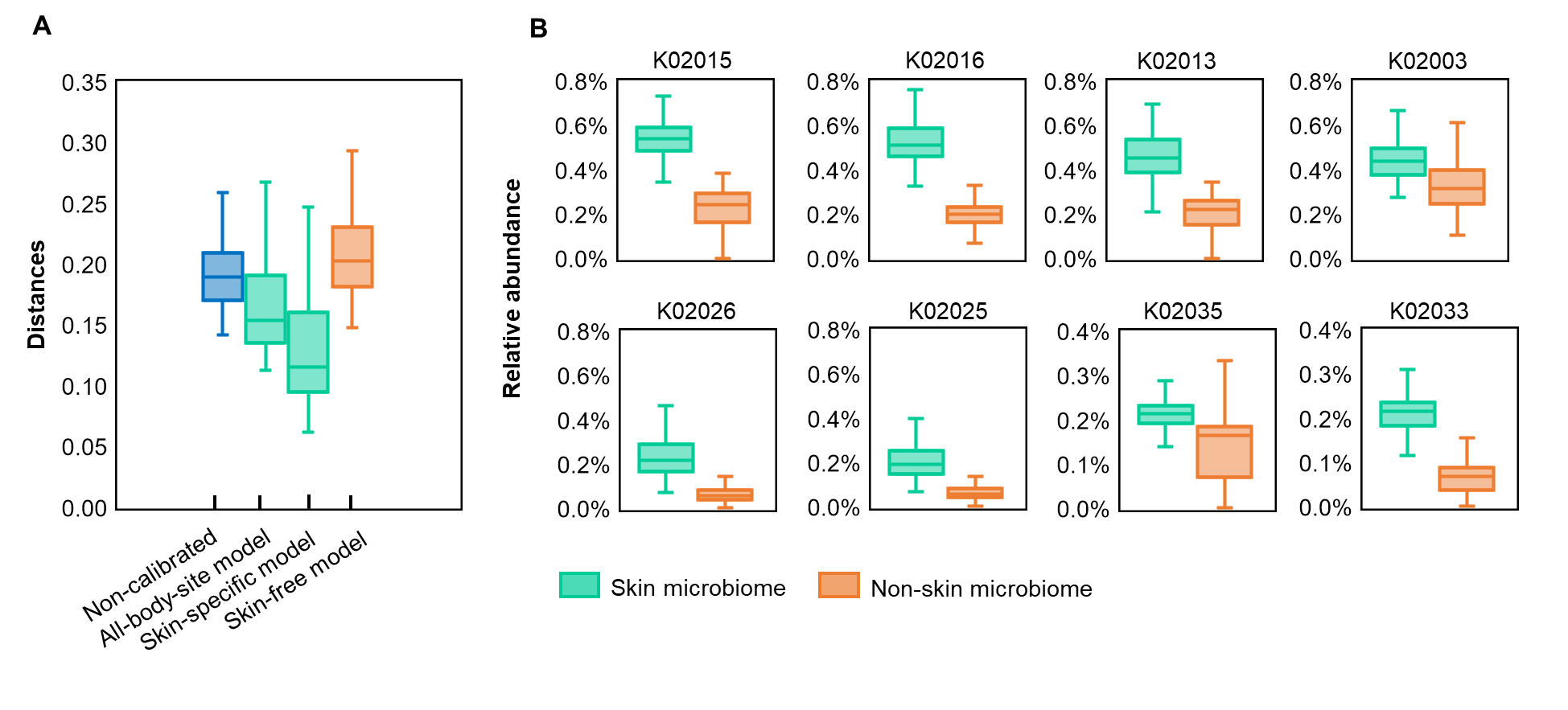
**

**Fig. S7. Calibration of skin amplicons using different habitat models.** (**A**) The Bray-Curtis distances between WGS and paired amplicon samples of non-calibrated, calibrated by model of 4-body-site training set, calibrated by skin-specific model and calibrated by skin-free model. (**B**) The functional features that were abundant or unique in skin samples but inadequate in skin-free model.


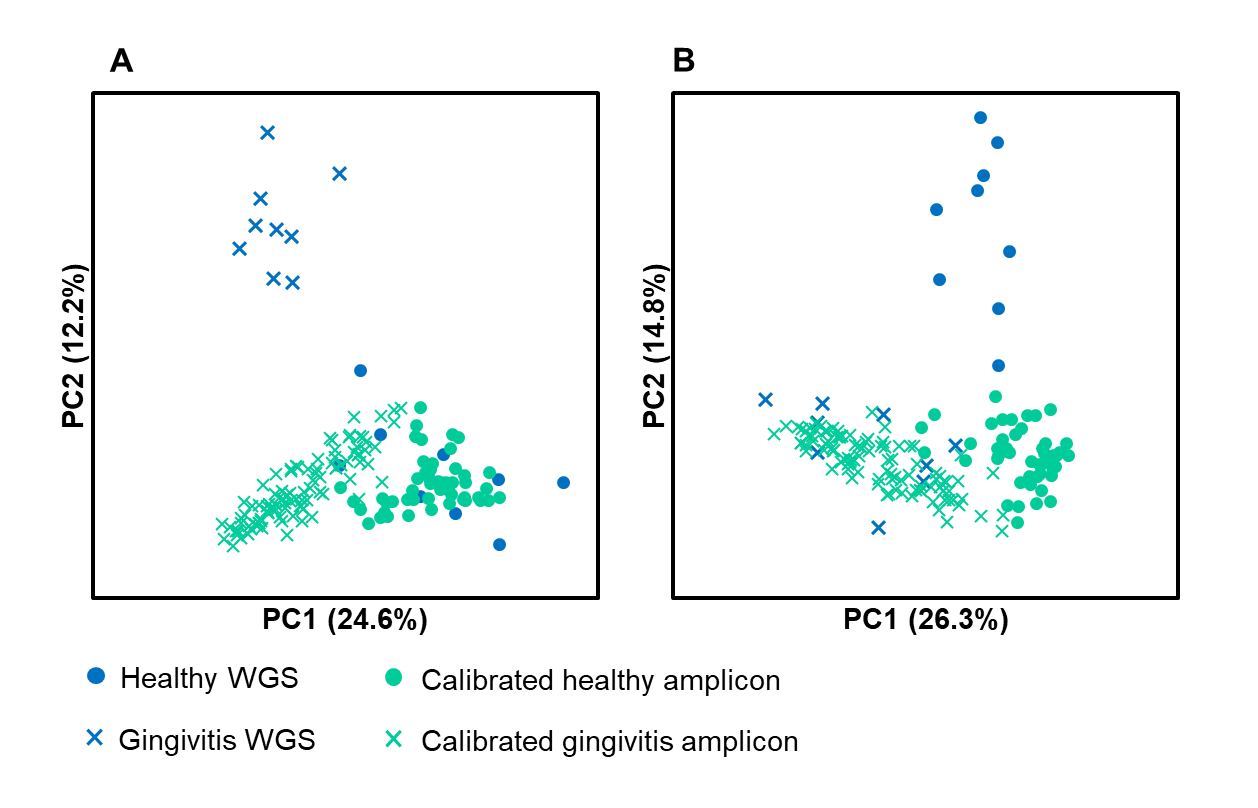


**Fig. S8. Calibration of amplicons for disease detection using status-specific models.** (**A**) Both healthy and disease amplicon samples shifted to the healthy WGS samples after the calibration with healthy-specific model. (**B**) Both healthy and disease amplicon samples shifted to the disease WGS samples after the calibration with disease-specific model.


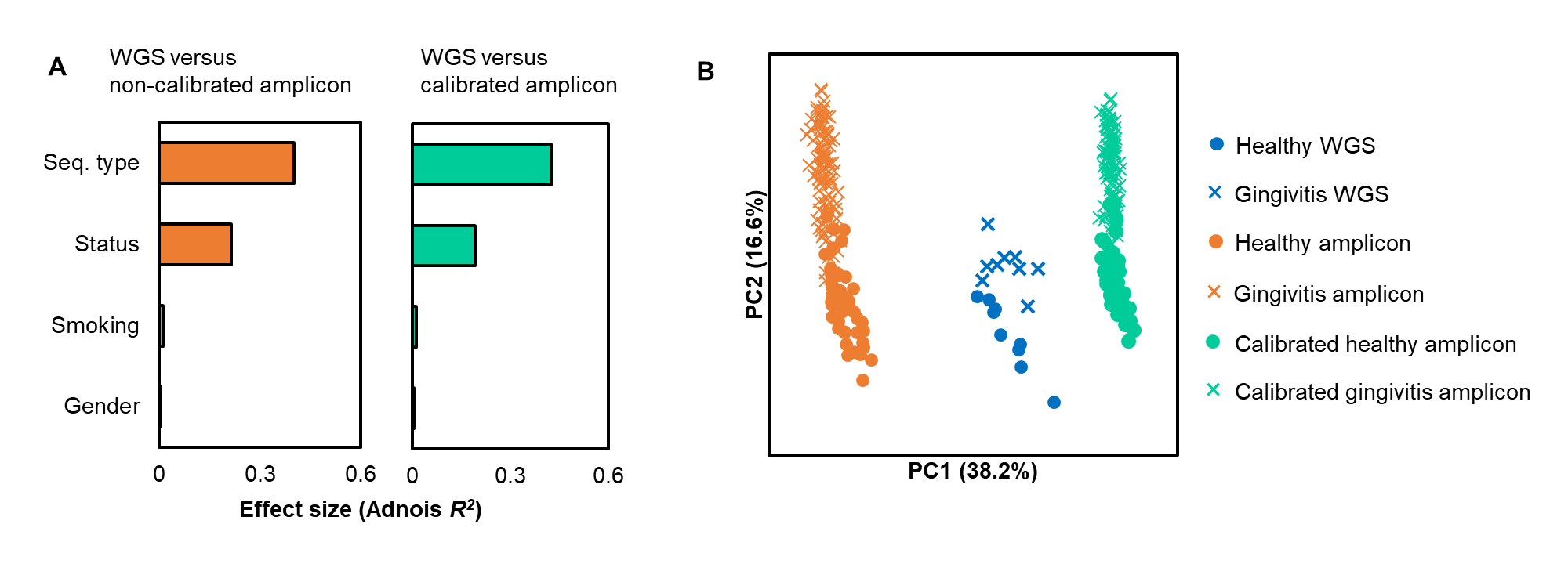


**Fig. S9. Calibration of amplicons using training samples that produced under inconsistent experiment protocols.** (**A**) Comparing the effect size of the sampling factors by Adonis test. (**B**) Beta-diversity pattern of calibrated amplicons shifted to unexpected direction.
